# A co-crystal between benzene and ethane: a potential evaporite material for Saturn’s moon Titan

**DOI:** 10.1107/S2052252516002815

**Published:** 2016-03-30

**Authors:** Helen E. Maynard-Casely, Robert Hodyss, Morgan L. Cable, Tuan Hoang Vu, Martin Rahm

**Affiliations:** a Bragg Institute, Australian Nuclear Science and Technology Organisation, Locked Bag 2001, Kirrawee DC, NSW 2232, Australia; b Jet Propulsion Laboratory, California Institute of Technology, 4800 Oak Grove Drive, Pasadena, CA 91109, USA; c Chemistry and Chemical Biology, Baker Laboratory, Cornell University, Ithaca, NY 14853, USA

**Keywords:** co-crystals, molecular crystallography, synchrotron powder diffraction, Titan, evaporite

## Abstract

Using synchrotron X-ray powder diffraction, the structure of a co-crystal between benzene and ethane formed *in situ* at cryogenic conditions has been determined, and validated using dispersion-corrected density functional theory calculations. The structure comprises a lattice of benzene molecules hosting ethane molecules within channels. Similarity between the intermolecular interactions found in the co-crystal and in pure benzene indicate that the C—H⋯π network of benzene is maintained in the co-crystal, however, this expands to accommodate the guest ethane molecules. The co-crystal has a 3:1 benzene:ethane stoichiometry and is described in the space group  with *a* = 15.977 (1) Å and *c* = 5.581 (1) Å at 90 K, with a density of 1.067 g cm^−3^. The conditions under which this co-crystal forms identify it is a potential that forms from evaporation of Saturn’s moon Titan’s lakes, an evaporite material.

## Abstract 2

A 3:1 stoichiometric co-crystal of benzene and ethane has been structurally characterized at 90 K using synchrotron powder X-ray diffraction following *in situ* crystal growth at 130 K. The conditions under which the co-crystal forms identify it as a potential evaporite material on the surface of Saturn’s moon Titan.

## 1. Introduction   

Crystallographic studies of benzene have a history of moving scientific understanding significantly forward. Kathleen Lonsdale’s pioneering work on the structure of hexa­methylbenzene showed the community that the benzene molecule was flat (Lonsdale, 1929 ▸), a study which, at least in part, laid the groundwork for molecular crystallography as we know it today. Later studies of the crystal structure of pure benzene (Cox *et al.*, 1958 ▸) showed that the flat benzene rings fit together like ‘six-tooth bevel gear wheels’ (Cox, 1932 ▸).

There is now growing interest in the structures of many simple hydrocarbons for a different reason. Titan, Saturn’s largest moon, has in recent years been revealed, largely by the on-going Cassini mission, to have a ‘hydrological’ cycle. Unlike the Earth’s hydrological cycle, Titan’s is not driven by water. The surface temperature on Titan is 91–95 K, and at these cryogenic temperatures the fluids that drive the cycle are small hydrocarbon molecules (Stofan *et al.*, 2007 ▸) such as methane and ethane, as well as dissolved dinitrogen. Lakes and seas observed on the surface of Titan contain a mixture of methane and ethane (Cordier *et al.*, 2009 ▸), which result from cloud formation and precipitation in the atmosphere. Additionally, there are a number of other small molecular species observed in the atmosphere, which are hypothesized to be present at Titan’s surface. These include organic molecules such as hydrogen cyanide, acetylene, ethylene, acetonitrile and benzene, formed photochemically from CH_4_ and N_2_ in the upper atmosphere (Vuitton *et al.*, 2008 ▸). In particular, benzene has been tentatively identified on the surface of Titan by the Huygens probe (Niemann *et al.*, 2005 ▸).

The observation of Titan’s hydrological cycle now encompasses lakes, seas, clouds and even rain of hydrocarbons – but it has been missing a vital piece. In light of the cycle observed at the surface, it is natural to ask whether Titan’s surface materials could produce deposits analogous to evaporites on Earth. Cassini imagery has collated evidence for possible evaporite deposits (Barnes *et al.*, 2011 ▸), but it remains a mystery as to what these materials could be made of. This is despite the important role that such materials would play in both the hydrological cycle and the surface chemistry of Titan.

In light of the discovery of Titan’s hydrological cycle, investigations have been undertaken to identify possible evaporite materials that would form on the surface. Recent results with Raman spectroscopy (Cable *et al.*, 2014 ▸; Vu *et al.*, 2014 ▸) on the interaction of small molecules under Titan surface conditions identified the formation of a possible co-crystal between benzene and ethane. Although spectroscopy and quantum-chemical calculations pointed to a specific local interaction between ethane and benzene molecules in the co-crystal, a crystallographic study is required to determine the structure unambiguously and consequently the composition of the co-crystal, as well as to ascertain its viability as an evaporite material on Titan.

## 2. Materials and methods   

### 2.1. Co-crystal growth and data collection   

Previous microscopic observations of the possible co-crystal showed that, on formation, the crystallite sizes were significantly reduced compared with that of frozen benzene (Vu *et al.*, 2014 ▸). Hence, we decided to pursue a powder X-ray diffraction study, using the high-resolution afforded by a synchrotron source. Approximately 2 µl of benzene (Sigma Aldrich 99.8%) was placed inside a 0.7 mm borosilicate capillary. The amount of benzene was tailored so that the length of the drop within the capillary was ∼ 2 mm. The capillary was then attached *via* a Swagelok fitting (Norby *et al.*, 1998 ▸) to a valve allowing the system to be closed, and mounted on the powder diffraction beamline at the Australian Synchrotron (Wallwork *et al.*, 2007 ▸), along with an Oxford Cryosystems cryostream (Cosier & Glazer, 1986 ▸) to control the sample temperature. The beamline was set up with λ = 0.826 (1) Å, verified by refinement of a pattern measured from NIST LaB6 (SRM 660*b*) powder standard. The X-ray beam from the synchrotron is vertically focused to a height of 1 mm, and the width of the beam was constrained to 3 mm with lead slits. A MYTHEN strip detector (Bergamaschi *et al.*, 2010 ▸) was used for all data collections.

**Figure 1:**
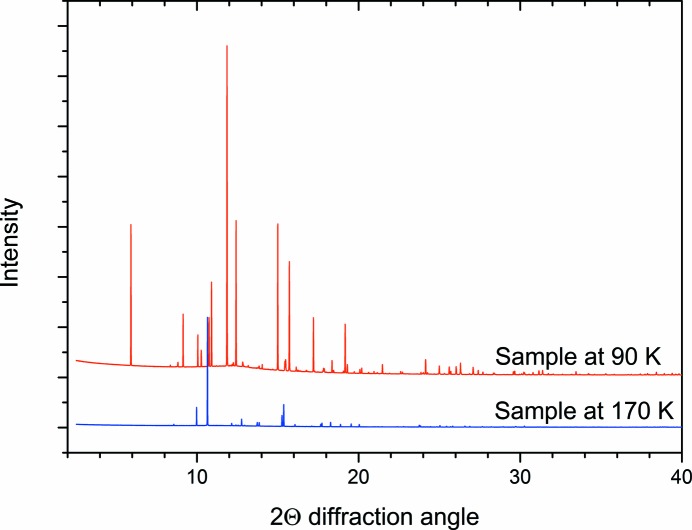
Comparison of the diffraction pattern of the sample at 90 and 170 K. The pattern at 170 K corresponds to pure benzene, while the pattern at 90 K contains peaks from both benzene and the benzene:ethane co-crystal.

The benzene within the capillary was frozen, and a diffraction pattern was measured at 170 K (Fig. 1 ▸; blue trace). The position of the capillary was then translated so that the edge of the frozen benzene was aligned with the centre of the X-ray beam, and the capillary system was attached to a bottle of ethane (Sigma Aldrich 99.9%). The temperature was reduced to 130 K, and ethane liquid was condensed adjacent to the frozen benzene. The interface was then monitored and cycling of the temperature between 130 and 90 K produced diffraction peaks additional to those of benzene, indicating the formation of the benzene:ethane co-crystal (Fig. 1 ▸; red trace). These additional peaks cannot be attributable to solid ethane, which freezes at 89 K. The formation of the co-crystal using this protocol was independently verified by Raman spectroscopic measurements (see the supporting information), in which a characteristic Raman feature at 2873 cm^−1^ (Vu *et al.*, 2014 ▸) appears on warming the system from 90 to 130 K. Once the co-crystal was formed at 130 K, the temperature was reduced to 90 K for a longer data acquisition. Acquiring the pattern at 90 K serves to minimize thermal motion in the crystal structure, and the longer acquisition time also revealed the signal from weaker peaks that may have been missed in the previous shorter acquisitions. The process of forming the co-crystal was repeated and the result was verified.

To investigate the thermal expansion and stability properties of the co-crystal, sequential patterns (collected over 120 s) were taken at 5 K intervals from 90 K to 150 K, then at 1 K intervals to 170 K. Previous results had suggested that the co-crystal would only be stable until ∼ 160 K (Vu *et al.*, 2014 ▸), and the smaller temperature intervals around this temperature allowed us to monitor the co-crystal decomposition.

### 2.2. Computational methods   

Once the structure of the co-crystal was established (as described in §3), periodic DFT calculations were performed using *VASP* (Version 5.3.5; Kresse & Furthmüller, 1996 ▸; Kresse & Joubert, 1999 ▸) to validate the result. The Perdew–Burke–Ernzerhof (PBE; Perdew *et al.*, 1996 ▸) GGA functional was used in combination with the DFT-D3 dispersion correction (Grimme *et al.*, 2010 ▸), standard projected augmented wave (PAW) potentials (Kresse & Joubert, 1999 ▸; Blöchl, 1994 ▸) and a plane-wave energy cutoff of 800 eV. Brillouin zone sampling was performed on a Monkhorst–Pack mesh, which spanned 3 × 3 × 9 *k*-points for the single unit cell. Energies and forces were converged to < 1 meV per atom.

Molecular calculations were performed using *ORCA* 3.03 (Neese, 2012 ▸). The gas-phase geometry of the {C_2_H_6_–(C_6_H_6_)_6_} cluster was optimized at the PBE-D3(BJ)/Def2-TZVPP level of theory. Vibrational analysis at the same level of theory showed the geometry to be dynamically stable, and provided zero-point energy corrections. Single-point energy calculations were performed using the second-order perturbation corrected ‘double hybrid’ density functional B2PLYP (Grimme, 2006 ▸) again together with the D3(BJ) dispersion correction (Grimme *et al.*, 2011 ▸) and in conjuction with the RIJCOSX approximation (Neese *et al.*, 2009 ▸) and auxillary def2-TZVPP/J and def2-TZVPP/C basis sets for separate coulomb and semi-numeric exchange integration. B2PLYP-D3(BJ) are expected to provide highly accurate energies. Specifically for intermolecular interactions, the reported mean absolute deviation is 1.2 kJ mol^−1^ (Grimme, 2011 ▸).

## 3. Results   

**Figure 2:**
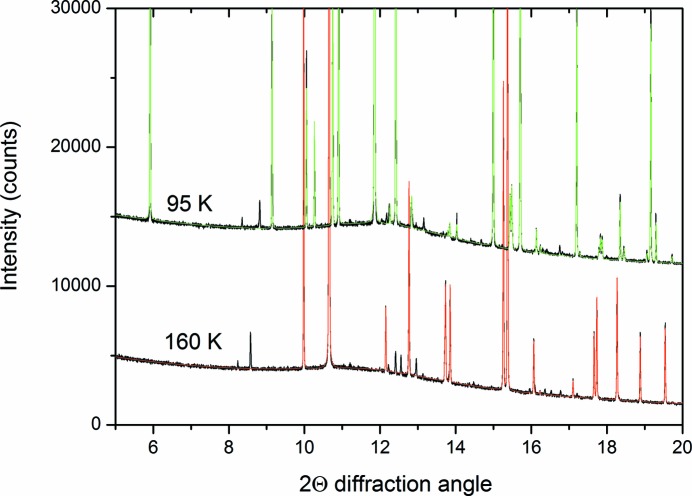
Partial plot of the patterns and Pawley fits of the co-crystal and benzene collected at 95 K (upper patterns displaced by 10 000 counts) and those of benzene collected at 160 K. The experimental data are plotted in black, the green calculated pattern is from a Pawley refinement of the benzene:ethane co-crystal and the red calculated pattern is a Pawley refinement of benzene. As explained in the text, there are a number of residual peaks, which persisted after the co-crystal had melted.

Working with the diffraction pattern taken at 90 K, the peaks not attributed to benzene were fitted with pseudo-Voigt functions using *TOPAS*4.1 (Coelho, 2008 ▸) then indexed to an *R*-centred unit cell with *a* = 15.977 (1), *c* = 5.581 (1) Å, which gives a volume of 1233.8 Å^3^. Pawley refinement (Pawley, 1981 ▸) in the space group *R*3 yielded *wR* = 0.031 and a goodness of fit (GoF) of 3.56. Systematic absences indicated either 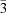 or 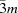 Laue groups, giving five possible space groups: *R*3, 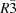, *R*32*, R*3*m* and 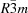. To begin our analysis of the crystal structure, the 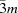 Laue class space groups (*R*32, *R*3*m* and 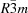) were ruled out as they would require a disordered structure to accommodate the benzene and ethane molecules. It was noted that there were still a number of small peaks that were not accounted for, as shown in Fig. 2 ▸. Further investigation indicated that these peaks persisted after the co-crystal melted in the first run but that they were not observed during the second run of the experiment. Hence, these peaks were judged not to belong to the co-crystal and were not considered further in the structure solution process. Attempts were made to index these additional peaks, but at most 13 of them were identified (from the pattern collected at 160 K), which proved insufficient to determine a unit cell for this potentially new phase.

The determined volume of the co-crystal unit cell placed constraints on the likely contents and stoichiometry of the structure. The density of ethane in its solid phase at 89 K is 0.669 g cm^−3^ (van Nes & Vos, 1978 ▸) and benzene at 90 K is 1.103 g cm^−3^ (Bacon *et al.*, 1964 ▸), so as a first assumption it was thought that the density of the co-crystal would lie between these values. Also considering the trigonal space group, there are only three possible combinations: a 1:1 benzene:ethane co-crystal with six formula units per unit cell, a 2:1 co-crystal with three formula units, and a 3:1 co-crystal also with three formula units. Unusual circumstances (*i.e.* the density of the co-crystal being lower than that of ethane or higher than that of benzene) could also have been pursued, if the structure solution using these potential contents had not been successful. To minimize the number of degrees of freedom during the structure solution process, the benzene and ethane molecules were constructed as rigid bodies, details of which are given in the supporting information. The arrangement of the rigid bodies was optimized against the 90 K pattern (Fig. 1 ▸), using a parallel-tempering algorithm within the Free Objects for Crystallography (*FOX*) program (Favre-Nicolin & Černý, 2002 ▸). The first co-crystallization run was used for the structure solution as the proportion of co-crystal formed (relative to the residual benzene) was higher. Prior to the minimization, a Le Bail refinement (Le Bail, 2005 ▸) of benzene was also undertaken in *FOX* to account for the peaks from this material in the pattern, the results of which were added to the parallel-tempering calculation. This process was undertaken for each of the three possible contents identified and in both potential space groups (*R*3 and 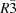).

**Figure 3:**
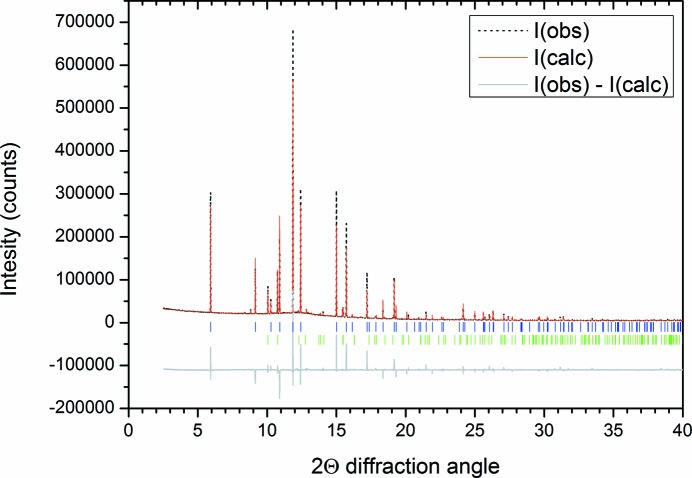
Rietveld fit of the 90 K pattern with the benzene and co-crystal structures (*R*_wp_ = 0.0741; GoF = 6.64). The grey line below the data indicates the difference between the observed and calculated patterns (offset by −100 000 counts for clarity). The blue tick marks indicate the positions of reflections from the co-crystal structure and green tick marks indicate the positions of reflections from benzene (Cox, 1932 ▸).

A viable crystal structure (judged by intermolecular distances and fit to the observed data) was obtained only for the 3:1 benzene:ethane co-crystal model, with three formula units in the unit cell. This structure was subjected to Rietveld refinement (Rietveld, 1969 ▸) using *TOPAS*4.1 (Coelho, 2008 ▸), giving the fit shown in Fig. 3 ▸. The parameters varied for this refinement were a scale factor, a background function, a zero error to account for displacement of the capillary, a single broad peak to account for the scattering of the borosilicate capillary, the lattice parameters for both of the phases, and the orientation and translation of the two rigid units used to build the co-crystal structure (details given in the supporting information). Diffraction from the pure benzene in the pattern was described with the structure determined by Cox *et al.* (1958 ▸), with the atoms fixed to these positions. Additionally, to account for the preferred orientation in the pure benzene phase, a fourth-order spherical harmonic model was added. Peak-shape parameters (Thompson–Cox–Hasting model; Thompson *et al.*, 1987 ▸) determined from the Pawley refinement were used, but fixed for the structural refinement, with crystallite size refined. Additional pseudo-Voigt peaks were also entered into the refinement to account for the intensity of the small peaks that were revealed at the indexing stage, as shown in Fig. 2 ▸. Atomic displacement parameters were constrained to be the same for the atoms within the benzene and ethane molecules, respectively. Table 1 ▸ lists the refined atomic coordinates for the co-crystal. The resultant density is 1.067 g cm^−3^ at 90 K, slightly less dense than solid benzene at 90 K (1.103 g cm^−3^). The determined H-atom positions are solely from geometric placement within the rigid bodies that were generated to solve the structure.

Fourier difference methods were used to determine whether there was any systematic electron density not accounted for by the structure. Structure factors were extracted from the refinement as presented in Fig. 3 ▸, and an |*F*_(obs)_| − |*F*_(calc)_| calculation was performed in the *VESTA* program (Momma & Izumi, 2008 ▸). The largest negative feature had a density of −0.02 e Å^−3^ and was situated between the ethane and benzene molecules; the largest positive feature was 0.05 e Å^−3^, which correlated with the C-atom positions within the ethane molecules. Given the small magnitude of the Fourier difference features, disordered models of the co-crystal were not explored.

The model for the 3:1 benzene:ethane co-crystal presented in Table 1 ▸ was also fitted (with the same refinement procedure) to the pattern collected from the second formation of the co-crystal at 90 K. Details of this fit are presented in the supporting information.

**Figure 4:**
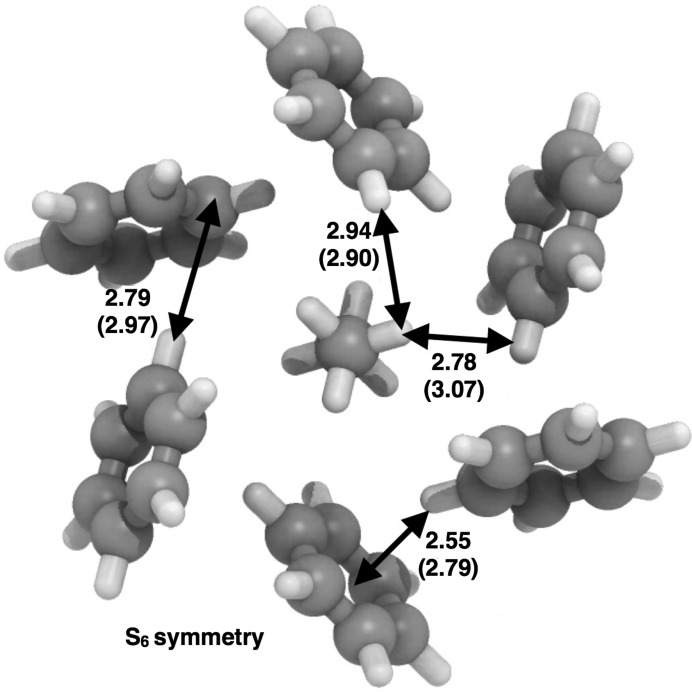
Optimized gas-phase geometry of a C_2_H_6_–(C_6_H_6_)_6_ cluster extracted from the co-crystal structure, along with selected distances (Å). Corresponding distances from the determined crystal structure are given in parentheses. The overall association energy of the cluster is estimated to be 108 kJ mol^−1^ near 0 K, and it is one likely seed structure in the growth of the final co-crystal.

Confirmation of the structure was sought from energy-minimization calculations. The periodic DFT calculations undertaken (results of which are detailed in the supporting information) include dispersion corrections and predict a structure in excellent agreement with the experimental determination of the benzene:ethane co-crystal structure. The unit-cell volume is 1234 Å^3^ from experiment and 1206 Å^3^ on minimization, differing by only 2%. Additionally, molecular calculations on a dispersion-bound C_2_H_6_–(C_6_H_6_)_6_ cluster (Fig. 4 ▸) predict it to be vibrationally stable also in isolation, with a quite substantial binding energy of 108 kJ mol^−1^ near 0 K.

**Figure 5:**
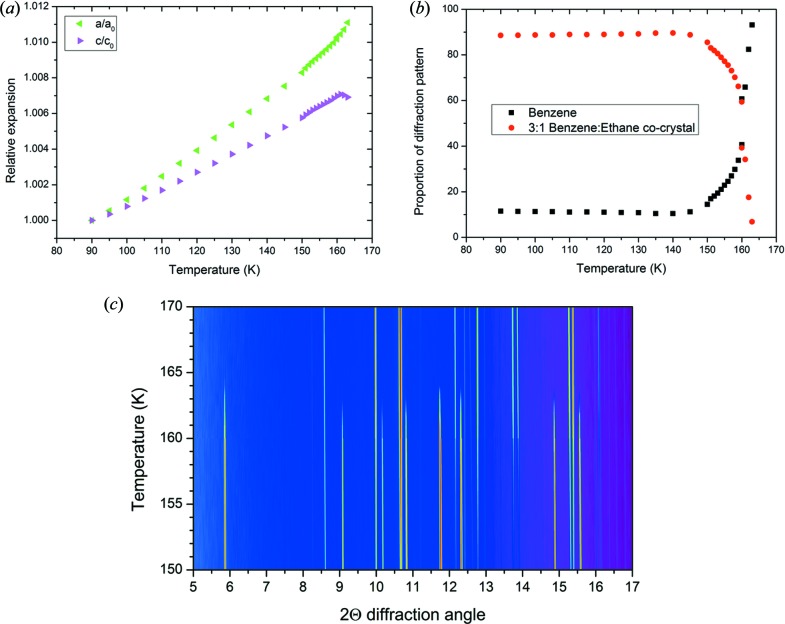
(a) Relative expansion of the *a* and *c* axes over the temperature range studied of the co-crystal, normalized to 90 K. (*b*) Relative proportion of the co-crystal and solid benzene in the patterns refined. (*c*) Plot of the diffraction data over the temperature range where the co-crystal decomposes.

Using the model of the co-crystal provided by the experimental structure solution process, each of the patterns from 90 to 165 K was refined and the lattice parameters and proportion of each phase (either benzene or co-crystal) were extracted from each pattern. Fig. 5 ▸(*a*) shows how the lattice parameters vary over the temperature range. From this, it can be seen that the co-crystal structure exhibits significant anisotropic thermal expansion, with the majority of the expansion occurring along the *a* and *b* axes.

The stability of the co-crystal structure is demonstrated by Fig. 5 ▸(*b*), which charts the relative proportion of the benzene and co-crystal refined in each diffraction pattern collected; the patterns measured between 150 and 170 K are presented in Fig. 5 ▸(*c*). The proportion of co-crystal to benzene in the patterns is steady at ∼ 89% co-crystal and ∼ 11% benzene from 90 to 145 K. Above 145 K, the proportion of the co-crystal in the refined pattern decreases monotonically. This is consistent with previous observations of the decomposition of the co-crystal at elevated temperatures (Cable *et al.*, 2014 ▸; Vu *et al.*, 2014 ▸). It is likely that, given sufficient time, above 145 K the co-crystal would decompose entirely without any increase of temperature.

## 4. Discussion   

**Figure 6:**
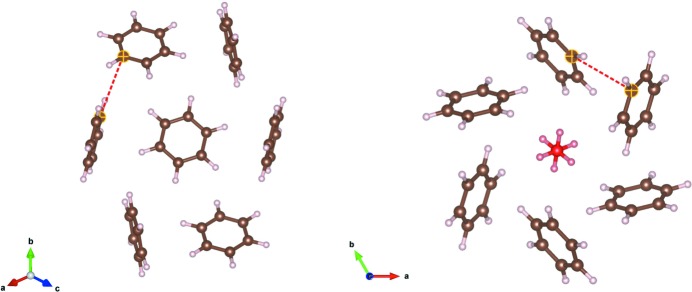
A section of the benzene crystal structure (Bacon *et al.*, 1964 ▸) (left) compared with the benzene:ethane co-crystal structure (right). The benzene structure is viewed down the [111] direction and the highlighted C⋯C distance is 3.85 Å. The benzene:ethane co-crystal structure is viewed down the *c* axis and the ethane molecule is coloured red. The highlighted C⋯C distance in the co-crystal is 3.80 (3) Å.

Inspection of the co-crystal over a number of unit cells shows that the structure is maintained by a network of C—H⋯π interactions very similar to those found in crystalline benzene. Fig. 6 ▸ compares the benzene crystal structure (as determined by Bacon *et al.*, 1964 ▸) with the benzene:ethane co-crystal. The ring of six benzene molecules around each of the ethane molecules is a feature that is ‘inherited’ from the pure benzene structure. In the co-crystal, the ethane molecules replace a quarter of the benzene molecules, and the benzene molecules move into a 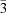 symmetry arrangement. This creates one-dimensional channels through the structure where the ethane molecules reside on the 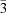 axes. Crucially, the benzene molecules maintains very similar C—H⋯π interactions (as shown by the distances highlighted in Fig. 6 ▸) compared to those in pure benzene.

**Figure 7:**
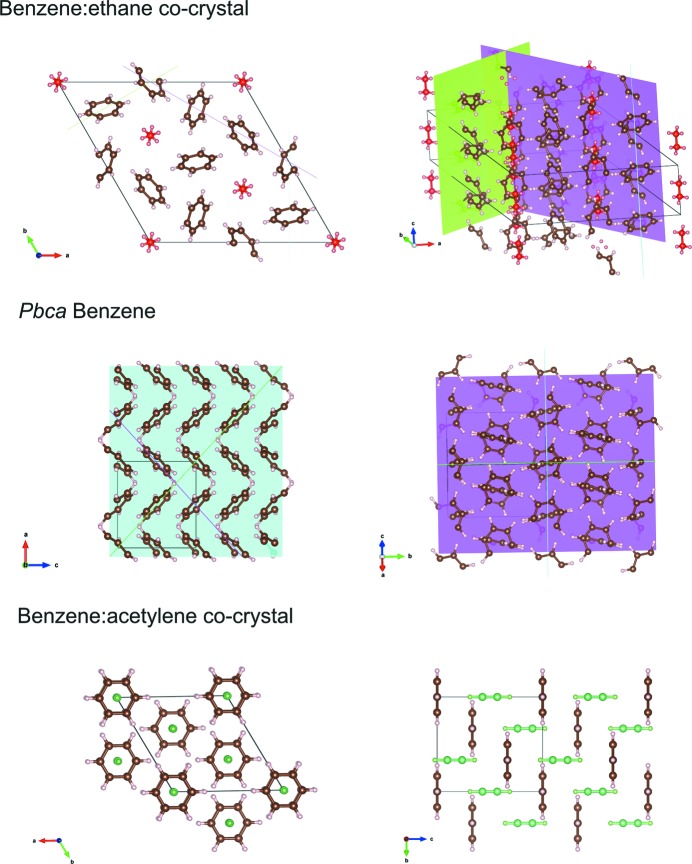
The packing of molecules and planes of C—H⋯π interactions across the benzene:ethane co-crystal, compared with that of benzene (Bacon *et al.*, 1964 ▸) and the benzene:acetylene co-crystal (Boese *et al.*, 2003 ▸). In the representation of the benzene:ethane co-crystal, the ethane molecules are coloured red. In the 1:1 benzene:acetylene co-crystal, the acetylene molecule are coloured green. The benzene:ethane and benzene structures have the planes of C—H⋯π interactions highlighted.

The similarities between the benzene:ethane co-crystal and pure benzene are explored further in Fig. 7 ▸. Additionally, the structure is compared with the only other co-crystal between benzene and a small hydrocarbon, namely a 1:1 benzene:acetylene co-crystal (Boese *et al.*, 2003 ▸). This shows how the C—H⋯π interactions between the benzene molecules create planes that run through both the benzene:ethane co-crystal and the benzene structure. The higher symmetry of the benzene:ethane co-crystal means that the benzene interactions create the channels parallel to the *c* axis where the ethane molecules are situated. The interactions in the benzene:ethane co-crystal and pure benzene are in stark contrast to the intermolecular interaction in the 1:1 benzene:acetylene co-crystal. Here, the benzene molecules are arranged so that they do not interact with each other, and instead are seen to form C—H⋯π interactions with the acetylene molecules which sit perpendicular to the benzene rings.

The similarity in the arrangement of benzene molecules in the co-crystal and in its pure form is echoed in the experimental Raman shifts observed by Vu *et al.* (2014 ▸), which are summarized in Table 2 ▸. The Raman modes of the benzene molecules show only modest shifts, of 0.3 cm^−1^ for ν_1_ and −3.1 and −1.8 cm^−1^ for ν_7_, indicating that the interactions of the molecules remain largely unchanged. This contrasts starkly with the observed changes in the Raman shifts of the ethane molecules in the co-crystal from the pure (liquid) form at 90 K. This, itself, is perhaps not surprising but the co-crystal’s ethane molecule Raman modes show a significant shift compared to that seen in solid ethane at 80 K [−4.4 cm^−1^ for ν_1_(*a*_1*g*_) and −6.7 cm^−1^ for ν_11_(*e_g_*)]. In the absence of the crystalline structure, Vu *et. al.* (2014 ▸) used electrostatic potential surface calculations of three benzene–ethane dimers to rationalize the origin of these Raman shifts. This previous work found the largest shift for the ethane molecules among the dimers studied to be −7 cm^−1^, arising from a monodentate interaction between the benzene and ethane molecules. These shifts can now be discussed in light of the co-crystal structure.

The first point arising is that the experimental Raman shifts are not due to specific C—H⋯π interactions between the ethane and benzene molecules in the co-crystal. The closest distance between an ethane H atom and the centre of a benzene ring is 4.15 (3) Å. This distance is large, compared with the C—H⋯π distance of 2.447 Å in the benzene:acetylene co-crystal (Boese *et al.*, 2003 ▸), the closest H(benzene)⋯benzene ring centroid distance in the co-crystal structure of 2.77 (3) Å, and the furthest C—H⋯π distance of 3.57 Å calculated by Vu *et al.* for a tridentate dimer between benzene and ethane. The next point is considering the orientation of the ethane molecules within the ‘channels’ of 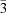 symmetry between the benzene rings (Fig. 6 ▸). It is interpreted that the ethane molecules are in a more constrained local environment within the co-crystal than in the monoclinic form of pure ethane (van Nes & Vos, 1978 ▸). This places more constraints on the motion of the ethane molecules, generating the noted experimental Raman shift.

The quantum mechanical calculations on the isolated subunit of ethane surrounded by six benzene molecules (Fig. 4 ▸) illustrates how effectively the arrangement of benzene molecules around each ethane molecules in the co-crystal maximizes the number of favourable C—H⋯π contacts beyond what is possible in pure benzene, or other co-crystals (Fig. 7 ▸). This specific coordination geometry allows for quite sizable dispersion (van der Waals) interactions (binding energy ∼ 108 kJ mol^−1^ of this cluster). Due to the relatively high association strength of this supramolecular entity, and its expected consequential persistence in solution at low temperature, we can speculate that it is one plausible seed structure in the build-up of the co-crystal.

**Figure 8:**
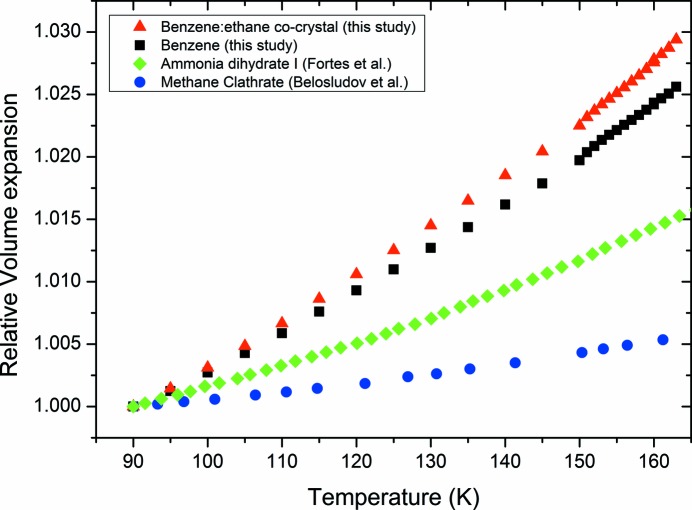
Comparison of the relative thermal expansion (normalized to 90 K) of the co-crystal, pure benzene (where the intermolecular interactions are dominated by C—H⋯π interactions), ammonia dihydrate I (Fortes *et al.*, 2003 ▸) and methane hydrate I (Belosludov *et al.*, 2002 ▸) (where the interactions in the crystal structures are dominated by hydrogen bonding).

The determined co-crystal structure can also explain the anisotropic thermal expansion noted in Fig. 5 ▸. This arises because the C—H⋯π interactions are aligned closer to the *a* and *b* axes in the co-crystal. Along the *c* axis, these chains of interactions interlock with each other (Fig. 7 ▸) and thereby restrict the expansion in this direction. As well as exhibiting anisotropic thermal expansion, Fig. 8 ▸ shows that the weak C—H⋯π interactions in the co-crystal lead to significantly higher relative thermal expansion compared to other possible Titan ‘minerals’, methane clathrate and ammonia dihydrate, which are dominated by hydrogen bonding (Fortes *et al.*, 2003 ▸; Belosludov *et al.*, 2002 ▸).

It seems likely that, given the channels the ethane molecules occupy within the benzene:ethane co-crystal, the other guest species could form similar co-crystals with benzene or partially substitute for ethane within the structure. Exchange of ethane with other linear hydrocarbons, or with HCN for example, is a target for future investigations, in the hope of further enriching our picture of Titan’s icy mineralogy.

## 5. Conclusions   

This study confirms the existence and structure of a 3:1 benzene:ethane co-crystal. The structure was solved using synchrotron powder X-ray diffraction and confirmed to be viable by dispersion-corrected DFT calculations. Conditions of the formation of this co-crystal suggest that it is a candidate evaporite material that will exist on the surface of Saturn’s moon Titan. It is, in fact, the first potential ‘cryogenic mineral’ to be identified where its intermolecular interactions are not dominated by hydrogen bonding. The co-crystal can therefore be presented as part of a new group of materials fused only by weak intermolecular interactions such as C—H⋯π, which could shape the surface of Titan. The structure of the co-crystal is substantially different from any known co-crystal of benzene. It is in significant contrast to a co-crystal formed between benzene and acetylene (Boese *et al.*, 2003 ▸), where the linear acetylene molecules align perpendicular to the benzene rings. The similarity of the interactions between the co-crystal and the structure of pure benzene show that the C—H⋯π network of benzene is maintained as a ‘host’, but expanded to allow the ethane ‘guest’ to situate within the channels that result from this network. We anticipate that this work will be followed by a number of other investigations charting the co-crystal formation and stability of other small molecular species that could become Titan’s ‘minerals’ and contribute to the understanding of the geology and potential habitability of this icy moon.

## Supplementary Material

Crystal structure: contains datablock(s) global, benzene_ethane_cocrystal, benzene, refinedPDpattern. DOI: 10.1107/S2052252516002815/bi5052sup1.cif

DFT-D optimized structure for the benzene:ethane co-crystal. DOI: 10.1107/S2052252516002815/bi5052sup2.txt

CCDC references: 1454031, 1454032

## Figures and Tables

**Table 1:** Atomic coordinates of the co-crystal model from the refinement presented in Fig. 3

Atom	*x*	*y*	*z*	*U*_iso_ (Å^2^)
C1*b*	0.547 (2)	0.000 (1)	−0.213 (2)	1.93 (3)
C2*b*	0.451 (2)	−0.070 (2)	−0.175 (3)	1.93 (3)
C3*b*	0.596 (2)	0.071 (2)	−0.038 (2)	1.93 (3)
H1*b*	0.417 (2)	−0.001 (2)	0.378 (2)	1.93 (3)
H2*b*	0.588 (2)	0.125 (2)	0.310 (3)	1.93 (3)
H3*b*	0.329 (2)	−0.127 (2)	0.067 (3)	1.93 (3)
C1*e*	0.000	0.000	0.363 (2)	2.2 (1)
H1*e*	−0.040 (2)	0.031 (2)	0.284 (2)	2.2 (1)

**Table 2:** Summary of experimental Raman shifts in the co-crystal compared with pure substances, from work by Vu *et al.* (2014 ▸) At 80 K, the crystal structure of ethane is assumed to have been the monoclinic phase of ethane, as described by van Nes & Vos (1978 ▸), rather than the plastic cubic phase that exists at ∼ 90 K

	ν_1_(*a*_1*g*_) CH_3_ *sym* stretch (cm^−1^)	ν_11_(*e_g_*) CH_3_ deform. stretch (cm^−1^)	ν_1_(*a*_1*g*_) CH *sym* stretch (cm^−1^)	ν_7_(*e*_2*g*_) CH *asym* stretch (cm^−1^)	ν_7_(*e*_2*g*_) CH *asym* stretch (cm^−1^)
Benzene in co-crystal at 90 K	–	–	3063.6	3040.6	3047.1
Solid benzene at 90 K	–	–	3063.3	3043.7	3048.9
Ethane in co-crystal at 90 K	2872.6	1454.8	–	–	–
Solid (monoclinic) ethane at 80 K	2877.0	1461.5	–	–	–
Liquid ethane at 90 K	2884.8	1467.1	–	–	–
